# (*E*)-3-(4-Meth­oxy­phen­yl)-1-[4-(piperidin-1-yl)phen­yl]prop-2-en-1-one

**DOI:** 10.1107/S1600536810026218

**Published:** 2010-07-14

**Authors:** Jerry P. Jasinski, Curtis J. Guild, B. Narayana, Prakash S. Nayak, H. S. Yathirajan

**Affiliations:** a Department of Chemistry, Keene State College, 229 Main Street, Keene, NH 03435-2001, USA; b Department of Studies in Chemistry, Mangalore University, Mangalagangotri 574 199, India; c Department of Studies in Chemistry, University of Mysore, Manasagangotri, Mysore 570 006, India

## Abstract

The piperidine ring in the title compound, C_21_H_23_NO_2_, is in a slightly distorted chair conformation. The dihedral angle between the two benzene rings is 5.6 (4)°. The dihedral angles between the propenone unit and the benzene and meth­oxy-substituted benzene rings are 5.6 (7) and 10.7 (8)°, respectively. Weak inter­molecular C—H⋯O hydrogen bonds and weak C—H⋯π inter­actions contribute to the stability of the crystal structure.

## Related literature

For the synthesis and biological evaluation of simple meth­oxy­lated chalcones as anti­cancer, anti-inflammatory and anti­oxidant agents, see: Bandgar *et al.* (2010 ▶). For anti-inflammatory chalcones, see: Nowakowska (2007 ▶). For related structures, see: Ahmad *et al.*(2010 ▶); Arai *et al.*(1994 ▶); Jasinski *et al.* (2010 ▶); Li *et al.* (1992 ▶); Patil *et al.* (2007 ▶); Shettigar *et al.* (2006 ▶). For standard bond lengths, see; Allen *et al.* (1987 ▶).
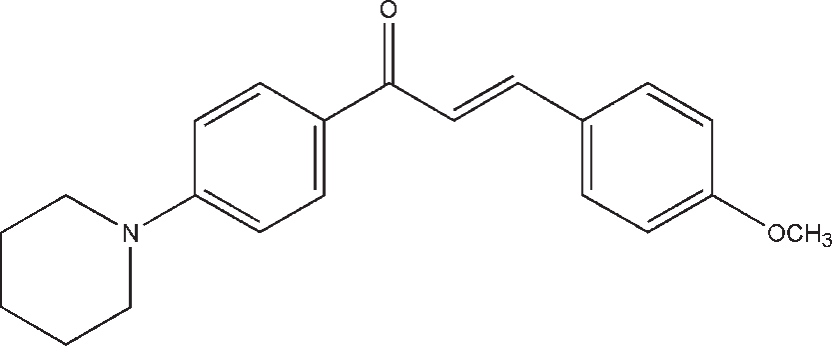


## Experimental

### 

#### Crystal data

C_21_H_23_NO_2_
*M* *_r_* = 321.40
Triclinic, 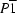

*a* = 6.0963 (11) Å
*b* = 10.985 (2) Å
*c* = 13.133 (3) Å
α = 74.188 (3)°
β = 88.674 (3)°
γ = 77.393 (3)°
*V* = 825.2 (3) Å^3^
*Z* = 2
Mo *K*α radiation
μ = 0.08 mm^−1^
*T* = 100 K
0.47 × 0.36 × 0.21 mm

#### Data collection

Bruker APEXII CCD diffractometer
Absorption correction: multi-scan (*SADABS*; Bruker, 2008 ▶) *T*_min_ = 0.962, *T*_max_ = 0.983
10674 measured reflections
4861 independent reflections
3562 reflections with *I* > 2σ(*I*)
*R*_int_ = 0.027

#### Refinement

*R*[*F* ^2^ > 2σ(*F* ^2^)] = 0.052
*wR*(*F* ^2^) = 0.151
*S* = 1.05
4861 reflections
218 parameters
H-atom parameters constrained
Δρ_max_ = 0.38 e Å^−3^
Δρ_min_ = −0.25 e Å^−3^

### 

Data collection: *APEX2* (Bruker, 2008 ▶); cell refinement: *SAINT* (Bruker, 2008 ▶); data reduction: *SAINT*; program(s) used to solve structure: *SHELXTL* (Sheldrick, 2008 ▶); program(s) used to refine structure: *SHELXTL*; molecular graphics: *SHELXTL*; software used to prepare material for publication: *SHELXTL* and *PLATON* (Spek, 2009 ▶).

## Supplementary Material

Crystal structure: contains datablocks global, I. DOI: 10.1107/S1600536810026218/lh5070sup1.cif

Structure factors: contains datablocks I. DOI: 10.1107/S1600536810026218/lh5070Isup2.hkl

Additional supplementary materials:  crystallographic information; 3D view; checkCIF report

## Figures and Tables

**Table 1:** Hydrogen-bond geometry (Å, °) *Cg*2 and *Cg*3 are the centroids of the C1–C6 and C15–C20 rings, respectively.

*D*—H⋯*A*	*D*—H	H⋯*A*	*D*⋯*A*	*D*—H⋯*A*
C20—H20⋯O2^i^	0.93	2.47	3.2105 (16)	137
C8—H8*A*⋯*Cg*3^ii^	0.97	2.66	3.623 (2)	171
C21—H21*A*⋯*Cg*2^iii^	0.96	2.90	3.823 (2)	162

Symmetry codes: (i) 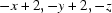; (ii) 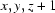; (iii) 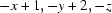.

**Fig. 1.**
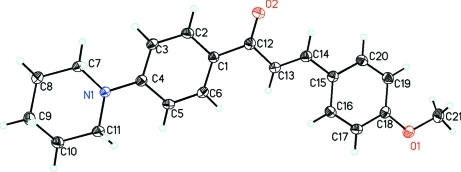
Molecular structure of the title compound showing the atom labeling scheme and 50% probability displacement ellipsoids.

**Fig. 2.**
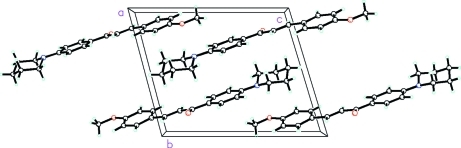
Packing of the title compound viewed along the a axis.

**Fig. 3.**
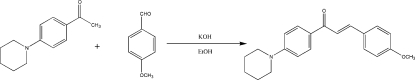
Reaction Scheme of C21H23N1O2,.

## supplementary crystallographic information

### Comment

The chalcone skeleton (1,3-diphenyl-2-propen-1-one) is a unique template that is associated with various biological activities. A review of anti- infective and anti-inflammatory chalcones is published (Nowakowska, 2007). The synthesis and biological evaluation of simple methoxylated chalcones as anticancer, anti-inflammatory and antioxidant agents is recently reported (Bandgar *et al.*, 2010). The crystal structures of some related chalcones containing methoxy substituents, *viz*., 4-bromo-4'-methoxy-chalcone (Li *et al.*, 1992), 4-bromo-4'-methoxychalcone (Arai *et al.*, 1994), 1-(4-bromophenyl)-3-(4-methoxyphenyl)prop-2-en-1-one (Shettigar *et al.*, 2006) and 1-(4-bromophenyl)-3-(3-methoxyphenyl) prop-2-en-1-one (Patil *et al.*, 2007), a monoclinic polymorph of 1-(4-chlorophenyl)-3- (4-methoxyphenyl)prop-2-en-1-one (Jasinski *et al.*, 2010) and (*E*)-3-(3-chlorophenyl)-1-(4-methoxyphenyl) prop-2-en-1-one (Ahmad *et al.*, 2010) have been reported. Hence in continuation with the synthesis and crystal structure of chalcones and their derivatives, this new chalcone containing the piperidine moiety was synthesized and its crystal structure is reported herein.

The title compound contains phenyl-4-methoxy and phenyl-4-piperidine groups on opposite sides of a 2-propen-1-one moiety (Fig. 1). Bond distances (Allen *et al.*,  1987) and angles are in normal ranges. The piperidine ring is in a slightly distorted chair conformation. The dihedral angles between the two benzene rings is 5.6 (4)°. The dihedral angles between the propenone moiety and the benzene and methoxy-benzene rings are 5.6 (7)° and 10.7 (8)°. A weak intermolecular hydrogen bond and weak intermolecular C—H···π-ring interactions contribute to the stability of crystal packing (Fig. 2).

### Experimental

A 30% KOH solution was added to a mixture of 1-[4-(piperidin-1-yl)phenyl] ethanone (0.01 mol, 2.03 g) and 4-methoxy benzaldehyde (0.01 mol, 1.36 g) in 50 ml of ethanol (Fig. 3). The mixture was stirred for an hour at room temperature and the precipitate was collected by filtration and purified by recrystallization from ethanol. The single-crystal was grown from 1:1 mixture of acetone and toluene by slow evaporation method and yield of the compound was 68% (m.p.391–394 K). Analytical data: Found (Calculated): C %: 78.41 (78.47); H%: 7.18 (7.21); N%: 4.33 (4.36).

### Refinement

All of the H atoms were placed in their calculated positions and then refined using the riding model with C—H = 0.93–0.97 Å, and with *U*_iso_(H) = 1.17–1.51*U*_eq_(C).

### Crystal data

**Table:**

C_21_H_23_NO_2_	*Z* = 2
*M**_r_* = 321.40	*F*(000) = 344
Triclinic, *P*1	*D*_x_ = 1.294 Mg m^−^^3^
Hall symbol: -P 1	Mo *K*α radiation, λ = 0.71073 Å
*a* = 6.0963 (11) Å	Cell parameters from 1958 reflections
*b* = 10.985 (2) Å	θ = 2.2–30.9°
*c* = 13.133 (3) Å	µ = 0.08 mm^−^^1^
α = 74.188 (3)°	*T* = 100 K
β = 88.674 (3)°	Block, red
γ = 77.393 (3)°	0.47 × 0.36 × 0.21 mm
*V* = 825.2 (3) Å^3^	

### Data collection

**Table:**

Bruker APEXII CCD diffractometer	4861 independent reflections
Radiation source: fine-focus sealed tube	3562 reflections with *I* > 2σ(*I*)
graphite	*R*_int_ = 0.027
ω scans	θ_max_ = 31.3°, θ_min_ = 1.6°
Absorption correction: multi-scan (*APEX2*; Bruker, 2008)	*h* = −8→8
*T*_min_ = 0.962, *T*_max_ = 0.983	*k* = −15→15
10674 measured reflections	*l* = −18→18

### Refinement

**Table:**

Refinement on *F*^2^	Primary atom site location: structure-invariant direct methods
Least-squares matrix: full	Secondary atom site location: difference Fourier map
*R*[*F*^2^ > 2σ(*F*^2^)] = 0.052	Hydrogen site location: inferred from neighbouring sites
*wR*(*F*^2^) = 0.151	H-atom parameters constrained
*S* = 1.05	*w* = 1/[σ^2^(*F*_o_^2^) + (0.0703*P*)^2^ + 0.1716*P*] where *P* = (*F*_o_^2^ + 2*F*_c_^2^)/3
4861 reflections	(Δ/σ)_max_ < 0.001
218 parameters	Δρ_max_ = 0.38 e Å^−^^3^
0 restraints	Δρ_min_ = −0.25 e Å^−^^3^

### Special details

**Table:**

Geometry. All e.s.d.'s (except the e.s.d. in the dihedral angle between two l.s. planes) are estimated using the full covariance matrix. The cell e.s.d.'s are taken into account individually in the estimation of e.s.d.'s in distances, angles and torsion angles; correlations between e.s.d.'s in cell parameters are only used when they are defined by crystal symmetry. An approximate (isotropic) treatment of cell e.s.d.'s is used for estimating e.s.d.'s involving l.s. planes.
Refinement. Refinement of *F*^2^ against ALL reflections. The weighted *R*-factor *wR* and goodness of fit *S* are based on *F*^2^, conventional *R*-factors *R* are based on *F*, with *F* set to zero for negative *F*^2^. The threshold expression of *F*^2^ > σ(*F*^2^) is used only for calculating *R*-factors(gt) *etc*. and is not relevant to the choice of reflections for refinement. *R*-factors based on *F*^2^ are statistically about twice as large as those based on *F*, and *R*- factors based on ALL data will be even larger.

### Fractional atomic coordinates and isotropic or equivalent isotropic displacement parameters (Å^2^)

**Table:**

	*x*	*y*	*z*	*U*_iso_*/*U*_eq_	
O1	0.23178 (16)	0.88147 (10)	−0.30730 (7)	0.0256 (2)	
O2	1.10171 (16)	0.82032 (10)	0.20063 (7)	0.0233 (2)	
N1	0.76112 (17)	0.61303 (11)	0.66686 (8)	0.0172 (2)	
C1	0.8660 (2)	0.75063 (12)	0.33849 (9)	0.0166 (2)	
C2	1.0241 (2)	0.74601 (13)	0.41547 (10)	0.0197 (3)	
H2	1.1548	0.7748	0.3940	0.024*	
C3	0.9928 (2)	0.70016 (13)	0.52239 (10)	0.0201 (3)	
H3	1.1021	0.6994	0.5710	0.024*	
C4	0.7989 (2)	0.65444 (12)	0.55956 (9)	0.0168 (2)	
C5	0.6400 (2)	0.65849 (13)	0.48138 (10)	0.0208 (3)	
H5	0.5103	0.6282	0.5022	0.025*	
C6	0.6729 (2)	0.70647 (13)	0.37468 (10)	0.0199 (3)	
H6	0.5630	0.7094	0.3256	0.024*	
C7	0.9508 (2)	0.59163 (14)	0.74157 (10)	0.0202 (3)	
H7A	1.0243	0.6641	0.7201	0.024*	
H7B	1.0594	0.5139	0.7385	0.024*	
C8	0.8785 (2)	0.57739 (14)	0.85498 (10)	0.0210 (3)	
H8A	0.7896	0.6599	0.8606	0.025*	
H8B	1.0111	0.5548	0.9015	0.025*	
C9	0.7413 (2)	0.47408 (13)	0.89082 (10)	0.0204 (3)	
H9A	0.8332	0.3896	0.8921	0.025*	
H9B	0.6895	0.4714	0.9616	0.025*	
C10	0.5413 (2)	0.50756 (13)	0.81348 (9)	0.0194 (3)	
H10A	0.4555	0.4405	0.8336	0.023*	
H10B	0.4440	0.5888	0.8171	0.023*	
C11	0.6156 (2)	0.51976 (13)	0.70057 (10)	0.0187 (3)	
H11B	0.6961	0.4354	0.6951	0.022*	
H11A	0.4836	0.5471	0.6532	0.022*	
C12	0.9153 (2)	0.79733 (12)	0.22544 (9)	0.0174 (2)	
C13	0.7416 (2)	0.81181 (12)	0.14373 (10)	0.0182 (3)	
H13	0.6061	0.7885	0.1641	0.022*	
C14	0.7769 (2)	0.85811 (12)	0.04061 (10)	0.0179 (3)	
H14	0.9108	0.8853	0.0243	0.021*	
C15	0.6283 (2)	0.87041 (12)	−0.04869 (9)	0.0174 (2)	
C16	0.4309 (2)	0.82206 (13)	−0.03711 (10)	0.0195 (3)	
H16	0.3856	0.7857	0.0304	0.023*	
C17	0.3037 (2)	0.82786 (13)	−0.12444 (10)	0.0202 (3)	
H17	0.1744	0.7948	−0.1154	0.024*	
C18	0.3679 (2)	0.88308 (13)	−0.22624 (10)	0.0198 (3)	
C19	0.5590 (2)	0.93488 (13)	−0.23992 (10)	0.0210 (3)	
H19	0.6008	0.9739	−0.3074	0.025*	
C20	0.6865 (2)	0.92747 (12)	−0.15117 (10)	0.0190 (3)	
H20	0.8146	0.9616	−0.1604	0.023*	
C21	0.3004 (3)	0.93119 (15)	−0.41233 (10)	0.0265 (3)	
H21A	0.3054	1.0207	−0.4238	0.040*	
H21B	0.1949	0.9240	−0.4624	0.040*	
H21C	0.4470	0.8823	−0.4214	0.040*	

### Atomic displacement parameters (Å^2^)

**Table:**

	*U*^11^	*U*^22^	*U*^33^	*U*^12^	*U*^13^	*U*^23^
O1	0.0261 (5)	0.0332 (6)	0.0188 (5)	−0.0104 (4)	−0.0024 (4)	−0.0061 (4)
O2	0.0210 (5)	0.0296 (5)	0.0216 (5)	−0.0118 (4)	0.0029 (4)	−0.0060 (4)
N1	0.0154 (5)	0.0228 (6)	0.0143 (5)	−0.0079 (4)	−0.0008 (4)	−0.0038 (4)
C1	0.0178 (6)	0.0159 (6)	0.0169 (6)	−0.0039 (4)	0.0006 (4)	−0.0054 (4)
C2	0.0153 (6)	0.0257 (7)	0.0190 (6)	−0.0080 (5)	0.0009 (4)	−0.0047 (5)
C3	0.0164 (6)	0.0268 (7)	0.0173 (6)	−0.0080 (5)	−0.0019 (5)	−0.0036 (5)
C4	0.0157 (6)	0.0182 (6)	0.0166 (6)	−0.0035 (4)	−0.0005 (4)	−0.0050 (5)
C5	0.0161 (6)	0.0295 (7)	0.0192 (6)	−0.0103 (5)	0.0014 (5)	−0.0065 (5)
C6	0.0186 (6)	0.0262 (7)	0.0168 (6)	−0.0085 (5)	−0.0009 (5)	−0.0064 (5)
C7	0.0172 (6)	0.0281 (7)	0.0171 (6)	−0.0097 (5)	−0.0012 (4)	−0.0054 (5)
C8	0.0211 (6)	0.0274 (7)	0.0167 (6)	−0.0094 (5)	−0.0003 (5)	−0.0066 (5)
C9	0.0206 (6)	0.0244 (7)	0.0154 (6)	−0.0059 (5)	0.0011 (5)	−0.0032 (5)
C10	0.0167 (6)	0.0240 (6)	0.0174 (6)	−0.0068 (5)	0.0010 (4)	−0.0034 (5)
C11	0.0166 (6)	0.0222 (6)	0.0183 (6)	−0.0072 (5)	−0.0004 (5)	−0.0048 (5)
C12	0.0201 (6)	0.0161 (6)	0.0163 (6)	−0.0044 (5)	0.0000 (5)	−0.0047 (5)
C13	0.0180 (6)	0.0190 (6)	0.0183 (6)	−0.0054 (5)	0.0003 (5)	−0.0052 (5)
C14	0.0177 (6)	0.0179 (6)	0.0185 (6)	−0.0039 (5)	0.0002 (5)	−0.0057 (5)
C15	0.0187 (6)	0.0154 (6)	0.0182 (6)	−0.0036 (4)	0.0007 (4)	−0.0048 (5)
C16	0.0201 (6)	0.0204 (6)	0.0177 (6)	−0.0057 (5)	0.0031 (5)	−0.0039 (5)
C17	0.0175 (6)	0.0213 (6)	0.0224 (6)	−0.0058 (5)	0.0015 (5)	−0.0057 (5)
C18	0.0201 (6)	0.0210 (6)	0.0182 (6)	−0.0031 (5)	−0.0016 (5)	−0.0062 (5)
C19	0.0226 (6)	0.0219 (6)	0.0177 (6)	−0.0064 (5)	0.0012 (5)	−0.0029 (5)
C20	0.0188 (6)	0.0181 (6)	0.0197 (6)	−0.0052 (5)	0.0020 (5)	−0.0038 (5)
C21	0.0305 (8)	0.0312 (8)	0.0177 (6)	−0.0068 (6)	−0.0013 (5)	−0.0063 (5)

### Geometric parameters (Å, °)

**Table:**

O1—C18	1.3711 (15)	C9—H9B	0.9700
O1—C21	1.4277 (16)	C10—C11	1.5194 (17)
O2—C12	1.2346 (15)	C10—H10A	0.9700
N1—C4	1.3882 (16)	C10—H10B	0.9700
N1—C7	1.4706 (15)	C11—H11B	0.9700
N1—C11	1.4713 (16)	C11—H11A	0.9700
C1—C6	1.3942 (17)	C12—C13	1.4779 (17)
C1—C2	1.3963 (16)	C13—C14	1.3408 (17)
C1—C12	1.4797 (17)	C13—H13	0.9300
C2—C3	1.3806 (17)	C14—C15	1.4575 (17)
C2—H2	0.9300	C14—H14	0.9300
C3—C4	1.4097 (17)	C15—C20	1.3948 (17)
C3—H3	0.9300	C15—C16	1.4071 (17)
C4—C5	1.4130 (16)	C16—C17	1.3785 (17)
C5—C6	1.3830 (17)	C16—H16	0.9300
C5—H5	0.9300	C17—C18	1.3949 (18)
C6—H6	0.9300	C17—H17	0.9300
C7—C8	1.5197 (17)	C18—C19	1.3920 (18)
C7—H7A	0.9700	C19—C20	1.3897 (17)
C7—H7B	0.9700	C19—H19	0.9300
C8—C9	1.5212 (18)	C20—H20	0.9300
C8—H8A	0.9700	C21—H21A	0.9600
C8—H8B	0.9700	C21—H21B	0.9600
C9—C10	1.5207 (17)	C21—H21C	0.9600
C9—H9A	0.9700		
			
C18—O1—C21	116.56 (10)	C11—C10—H10B	109.3
C4—N1—C7	117.45 (10)	C9—C10—H10B	109.3
C4—N1—C11	117.85 (10)	H10A—C10—H10B	108.0
C7—N1—C11	113.84 (10)	N1—C11—C10	112.56 (10)
C6—C1—C2	116.73 (11)	N1—C11—H11B	109.1
C6—C1—C12	124.41 (11)	C10—C11—H11B	109.1
C2—C1—C12	118.84 (11)	N1—C11—H11A	109.1
C3—C2—C1	122.16 (11)	C10—C11—H11A	109.1
C3—C2—H2	118.9	H11B—C11—H11A	107.8
C1—C2—H2	118.9	O2—C12—C13	121.02 (11)
C2—C3—C4	121.43 (11)	O2—C12—C1	119.88 (11)
C2—C3—H3	119.3	C13—C12—C1	119.07 (11)
C4—C3—H3	119.3	C14—C13—C12	120.87 (12)
N1—C4—C3	121.98 (11)	C14—C13—H13	119.6
N1—C4—C5	121.74 (11)	C12—C13—H13	119.6
C3—C4—C5	116.22 (11)	C13—C14—C15	127.06 (12)
C6—C5—C4	121.47 (12)	C13—C14—H14	116.5
C6—C5—H5	119.3	C15—C14—H14	116.5
C4—C5—H5	119.3	C20—C15—C16	117.66 (11)
C5—C6—C1	121.97 (11)	C20—C15—C14	119.35 (11)
C5—C6—H6	119.0	C16—C15—C14	122.93 (11)
C1—C6—H6	119.0	C17—C16—C15	120.88 (12)
N1—C7—C8	112.76 (10)	C17—C16—H16	119.6
N1—C7—H7A	109.0	C15—C16—H16	119.6
C8—C7—H7A	109.0	C16—C17—C18	120.37 (12)
N1—C7—H7B	109.0	C16—C17—H17	119.8
C8—C7—H7B	109.0	C18—C17—H17	119.8
H7A—C7—H7B	107.8	O1—C18—C19	124.56 (12)
C7—C8—C9	112.12 (10)	O1—C18—C17	115.48 (11)
C7—C8—H8A	109.2	C19—C18—C17	119.96 (11)
C9—C8—H8A	109.2	C20—C19—C18	119.01 (12)
C7—C8—H8B	109.2	C20—C19—H19	120.5
C9—C8—H8B	109.2	C18—C19—H19	120.5
H8A—C8—H8B	107.9	C19—C20—C15	122.08 (12)
C10—C9—C8	108.32 (10)	C19—C20—H20	119.0
C10—C9—H9A	110.0	C15—C20—H20	119.0
C8—C9—H9A	110.0	O1—C21—H21A	109.5
C10—C9—H9B	110.0	O1—C21—H21B	109.5
C8—C9—H9B	110.0	H21A—C21—H21B	109.5
H9A—C9—H9B	108.4	O1—C21—H21C	109.5
C11—C10—C9	111.56 (10)	H21A—C21—H21C	109.5
C11—C10—H10A	109.3	H21B—C21—H21C	109.5
C9—C10—H10A	109.3		
			
C6—C1—C2—C3	0.01 (19)	C6—C1—C12—O2	−171.55 (13)
C12—C1—C2—C3	−178.13 (12)	C2—C1—C12—O2	6.44 (18)
C1—C2—C3—C4	0.5 (2)	C6—C1—C12—C13	6.57 (19)
C7—N1—C4—C3	−12.83 (18)	C2—C1—C12—C13	−175.44 (11)
C11—N1—C4—C3	−154.97 (12)	O2—C12—C13—C14	−4.24 (19)
C7—N1—C4—C5	170.04 (11)	C1—C12—C13—C14	177.66 (11)
C11—N1—C4—C5	27.90 (18)	C12—C13—C14—C15	176.00 (11)
C2—C3—C4—N1	−177.33 (12)	C13—C14—C15—C20	176.56 (12)
C2—C3—C4—C5	−0.05 (19)	C13—C14—C15—C16	−6.3 (2)
N1—C4—C5—C6	176.42 (12)	C20—C15—C16—C17	1.80 (19)
C3—C4—C5—C6	−0.9 (2)	C14—C15—C16—C17	−175.43 (12)
C4—C5—C6—C1	1.4 (2)	C15—C16—C17—C18	−0.6 (2)
C2—C1—C6—C5	−0.95 (19)	C21—O1—C18—C19	3.18 (19)
C12—C1—C6—C5	177.09 (12)	C21—O1—C18—C17	−176.80 (11)
C4—N1—C7—C8	166.66 (11)	C16—C17—C18—O1	178.77 (11)
C11—N1—C7—C8	−49.73 (15)	C16—C17—C18—C19	−1.2 (2)
N1—C7—C8—C9	53.10 (15)	O1—C18—C19—C20	−178.31 (12)
C7—C8—C9—C10	−55.90 (15)	C17—C18—C19—C20	1.7 (2)
C8—C9—C10—C11	56.61 (14)	C18—C19—C20—C15	−0.4 (2)
C4—N1—C11—C10	−165.90 (11)	C16—C15—C20—C19	−1.33 (19)
C7—N1—C11—C10	50.64 (15)	C14—C15—C20—C19	176.00 (12)
C9—C10—C11—N1	−54.75 (15)		

### Hydrogen-bond geometry (Å, °)

**Table:**

Cg2 and Cg3 are the centroids of the C1–C6 and C15–C20 rings, respectively.

**Table:**

*D*—H···*A*	*D*—H	H···*A*	*D*···*A*	*D*—H···*A*
C20—H20···O2^i^	0.93	2.47	3.2105 (16)	137
C8—H8A···Cg3^ii^	0.97	2.66	3.623 (2)	171
C21—H21A···Cg2^iii^	0.96	2.90	3.823 (2)	162

Symmetry codes: (i) −*x*+2, −*y*+2, −*z*; (ii) *x*, *y*, *z*+1; (iii) −*x*+1, −*y*+2, −*z*.
